# Chemical Constituents and Pharmacological Effects of *Camellia oleifera* Fruits: A Review

**DOI:** 10.3390/molecules30193965

**Published:** 2025-10-02

**Authors:** Bing Xu, A-Nan Du, Tian-Zhi Liu, Ping-Hui Wei, Bo-Rong Zhu, Kai Chen, Lin Shi

**Affiliations:** 1 Shangrao Innovation Institute of Agricultural Technology, Shangrao Normal University, Shangrao 334001, China; 2 College of Food Science, Shenyang Agricultural University, Shenyang 110866, China; 3 Jiangxi Xin Zhongye Camellia Industry, Shangrao 334599, China

**Keywords:** *Camellia oleifera*, fruit, chemical constituents, pharmacological effects, triterpenoids, favonoids

## Abstract

*Camellia oleifera*, a member of the Theaceae family and belonging to the *Camellia* Linn species, is a plant utilized for edible oil production and medicinal value. Its fruit is abundant in various bioactive compounds, including triterpene saponins, flavonoids, lignans, fatty acids, sterols, polysaccharides, and numerous other chemical constituents. Among these, triterpene saponins and flavonoids serve as the primary active ingredients. The pharmacological effects of *C. oleifera* fruits are diverse, encompassing anti-tumor properties, cardiovascular and cerebrovascular protection, anti-inflammatory, antioxidant activity, lipid-lowering capability, anti-fungal property, and neuroprotective function. In recent years, this area has garnered significant attention from scholars both domestically and internationally. This article reviews the chemical constituents and pharmacological effects of *C. oleifera* fruits, aiming to provide a comprehensive reference for further research and development. Additionally, it offers a scientific foundation and innovative insights for clinical applications and the identification of relevant bioactive components.

## 1. Introduction

*Camellia oleifera* Abel. (Theaceae family), a high-quality edible oil plant endemic to China, is recognized as one of the four major woody oil plants globally, alongside olive, oil palm, and coconut [1]. The seeds of *C. oleifera* are distinguished by their high oil content, with a significant proportion of unsaturated fatty acids, predominantly oleic acid (about 80%) [2].

The fruit shells, seeds, and tea seed cakes (defatted seeds) of *C. oleifera* fruits contain various components, including triterpene saponins, flavonoids, lignans, tannins, fatty acids, and polysaccharides [3,4,5]. The main active constituents are triterpene saponins and flavonoids. Tea saponin, a pentacyclic triterpene saponin and glycoside molecule, exhibits excellent natural surfactant properties and antibacterial, antitumor, anti-inflammatory, antioxidant, and other properties [6,7,8,9,10]. Therefore, it is widely used in the pharmaceutical, cosmetic, functional food, and pesticide industries, among other sectors [11,12,13,14]. 

The oil derived from the mature seeds of *C. oleifera* is known as “Eastern olive oil” and is rich in nutrients, clear in color, and aromatic in flavor. The 2020 edition of the Chinese *Pharmacopoeia* lists this premium edible vegetable oil as a foundation for ointments and a raw ingredient for tea oil for injection [15]. Tea seed cake also has therapeutic benefits. It can remove heat, encourage blood flow, eliminate blood stasis, and relieve pain. China is the original home and distribution center of *C. oleifera*, with a long history of cultivation and abundant genetic resources. However, the majority of processed goods made from it are restricted to primary goods like tea seed oil. The underdeveloped production and processing of by-products, such as tea seed cakes and fruit shells, limit the possibility for sustainable industry expansion and lead to wasteful resource management.

**Figure 1:**
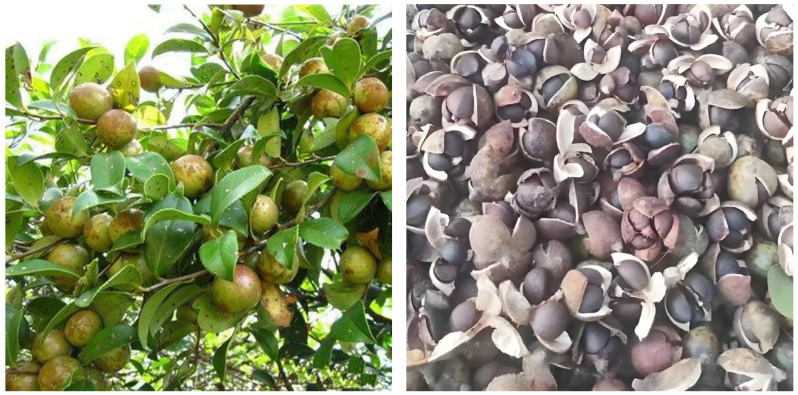
The fruits of *C. oleifera*.

Human health and quality of life are still seriously threatened by cancer, hyperlipidemia, and inflammatory chronic diseases. Research on new drugs is now focused on finding natural compounds that contain multi-target synergistic inhibitors that are safer, more effective, and less expensive [16,17]. Nowadays, dual-use products for food and medicine play a significant role in the prevention and management of chronic diseases and age-related conditions [18]. In this paper, we reviewed the primary chemical constituents and pharmacological effects of *C. oleifera* fruits (Figure 1), thereby providing a scientific foundation and strategic insights for the further development and utilization of this plant, particularly in functional foods and pharmaceutical applications.

## 2. Phytochemical Composition

### 2.1. Triterpenoids and Triterpenoid Saponins

**Figure 2:**
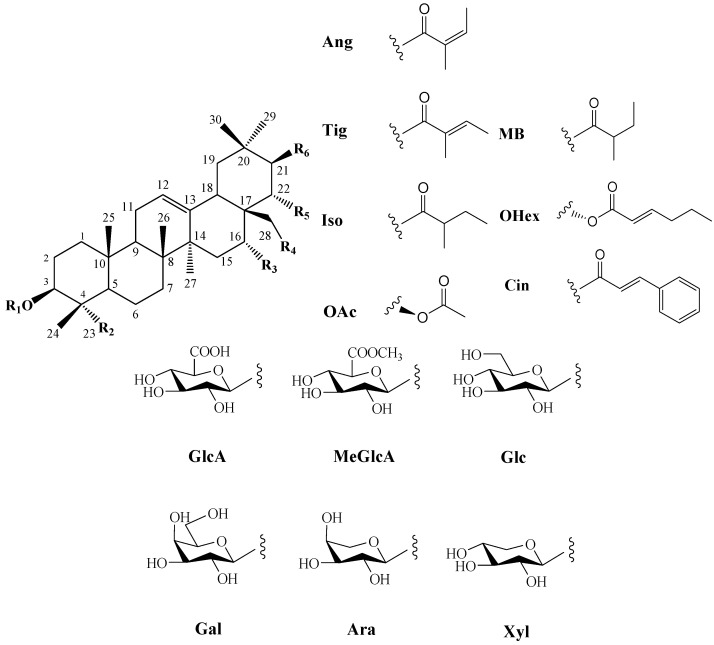
Structures of triterpenoids in *C. oleifera* fruit. **R_1_**–**R_6_** are substituent groups.

**Figure 3:**
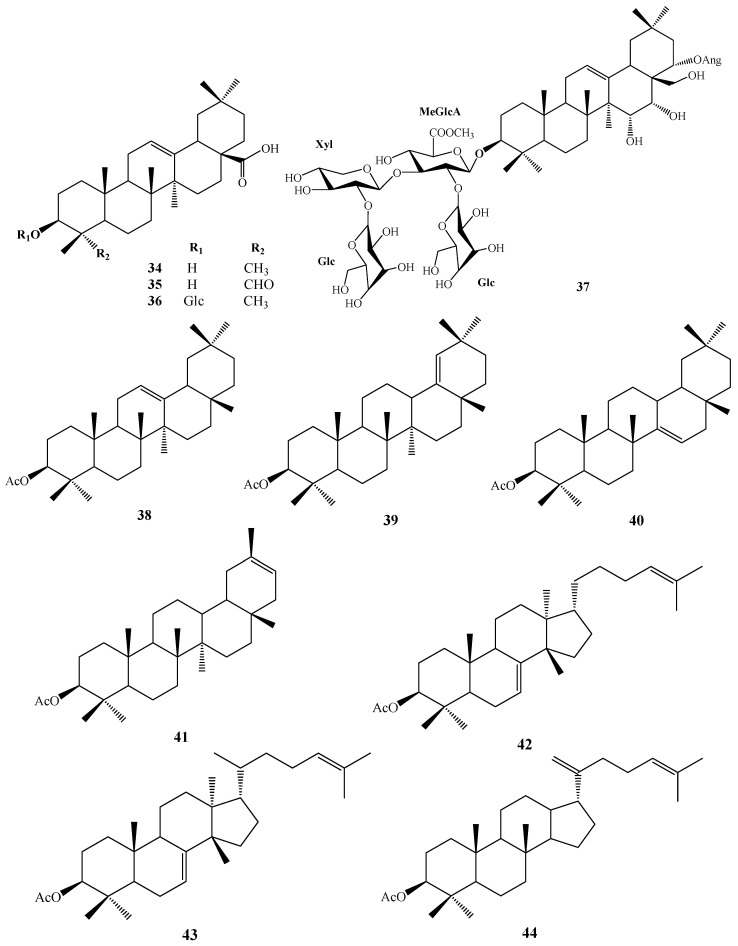
Structures of **34**–**44**.

In 1930, Aoyama first isolated Camellia saponin from tea tree seeds and named it “Theasaponin”, which belongs to the triterpenoid saponins [19]. *C. oleifera* fruit also has many saponin components, primarily oleanane-type pentacyclic triterpene saponins. Figure 2 depicts the structural mother nucleus of the saponin compounds. This paper summarizes 44 triterpenoids and triterpenoid saponins that have been isolated and reported [20,21,22,23,24,25,26,27,28,29,30,31,32,33]. The following are 33 of them listed in Table 1. The chemical structures of oleanolic acid (**34**), quillaic acid (**35**), oleanolic acid 3-O-*β*-D-glucoside (**36**) [33], camelliasaponin Ab (**37**) [30], *β*-amyrin acetate (**38**), germanol acetate (**39**), taraxerol acetate (**40**), *Ψ*-taraxasterol acetate (**41**), butyrospermol acetate (**42**), kansonidiol acetate (**43**), and damadienol acetate (**44**) [34] are presented in Figure 3.

The mother nuclei of these triterpenoids are predominantly pentacyclic triterpenoids of the oleanane type, which contain multiple active substitution sites primarily located at carbon positions C-3, C-16, C-21, C-22, C-23, and C-28. The molecular structure of most saponins consists of three distinct components: aglycones, sugar moieties, and organic acids [35]. Sugar substituents are mainly attached at the C-3 position, with the sugar chain composed predominantly of pentoses such as arabinose (Ara) and xylose (Xyl), as well as hexoses including glucose (Glc) and galactose (Gal). In addition, glucuronic acid (GlcA) and methylglucuronic acid (MeGlcA) are also commonly observed. These sugar units typically form oligosaccharide chains that are covalently linked to the aglycone, thereby generating triterpenoid saponins. The associated organic acids—such as acetic acid, angelic acid, isovaleric acid, hexenoic acid, 2-methylbutyric acid, tiglic acid, and cinnamic acid—can undergo esterification with specific hydroxyl groups on the saponin molecules, yielding ester derivatives.

### 2.2. Flavonoids and Their Glycosides

**Figure 4:**
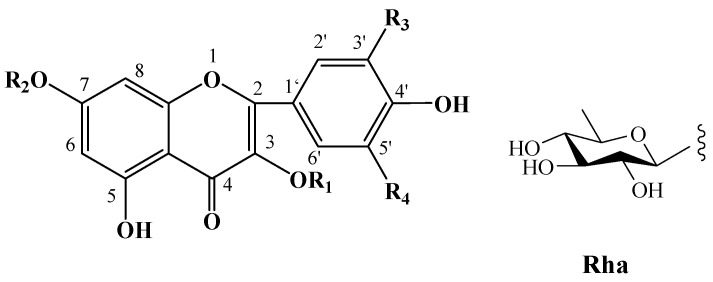
Structures of flavonoids in *C. oleifera* fruits. **R_1_**–**R_4_** are substituent groups.

**Figure 5:**
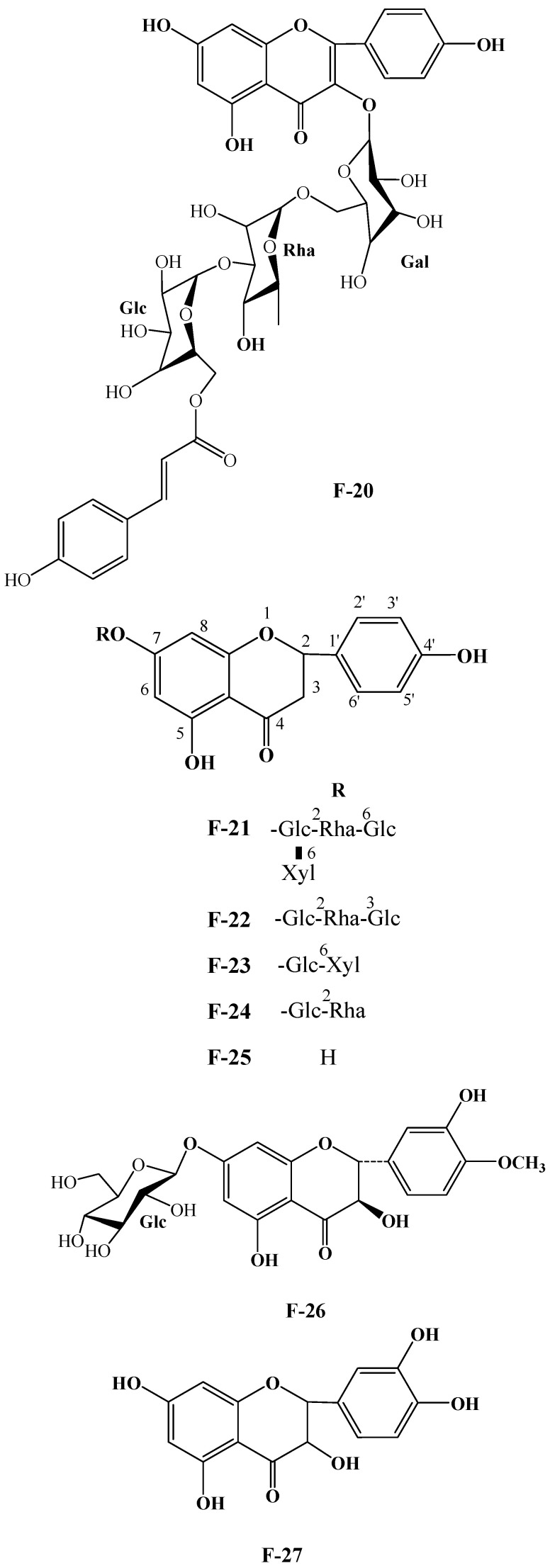

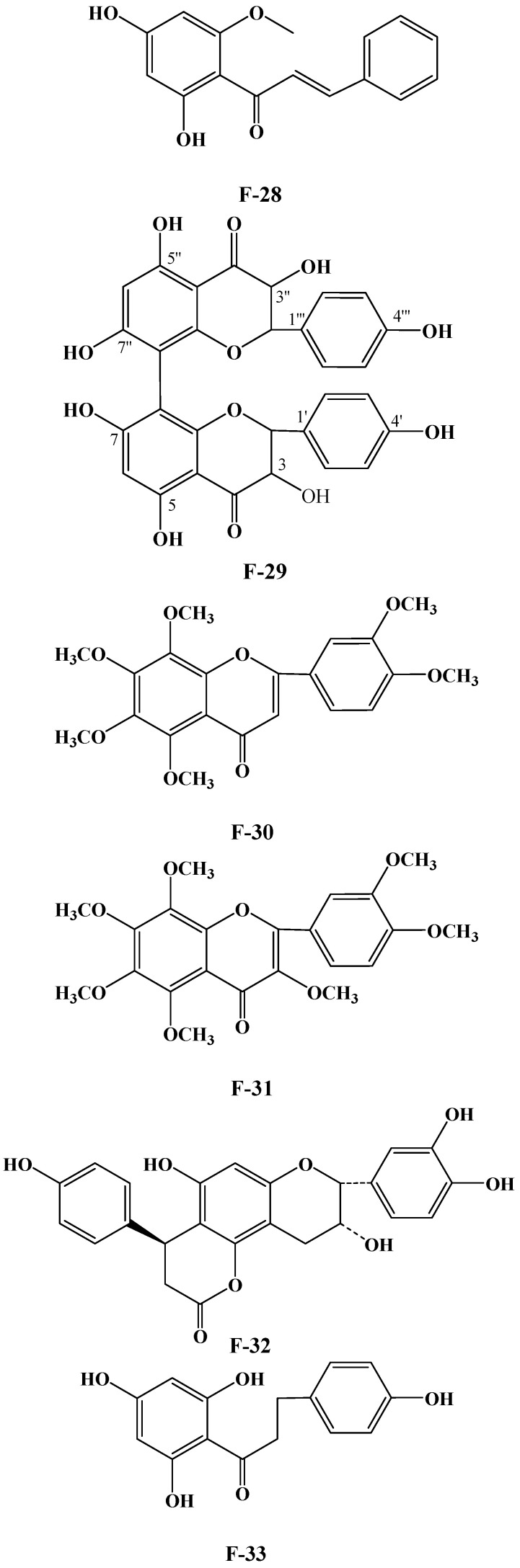
Structures of **F-20**, **F-21~25**, **F-26**, **F-27**, **F-28**, **F-29**, **F-30**, **F-31**, **F-32,** and **F-33**.

Numerous plants contain flavonoids, which are significant secondary metabolites. According to research, *C. oleifera* fruits have a comparatively large and diverse concentration of these components, primarily flavonols and their glycosides. Its structural mother nucleus is mainly composed of flavonols such as quercetin and kaunferol [36]. Arabinose (Ara), xylose (Xyl), glucose (Glc), rhamnose (Rha), and galactose (Gal) make up the majority of the sugar chain. The structural mother nucleus of the flavonoids found in *C. oleifera* fruit is displayed in Figure 4. The following are 19 of them listed in Table 2. The chemical structures of kaempferol-3-*O*-[6-trans-p-coumarol]-*β*-D-glucopyranosyl-(1 → 3)-*α*-L-rhamnopyranosyl-(1 → 6)-*O*-*β*-D-galactopyranoside (**F-20**) [37], naringenin-7-*O*-[*β*-D-xylopyranosyl-(1 → 6)][*β*-D-glucopyranosyl(1 → 3)-*α*-L-rhamnopyranosyl(1 → 2)-*O*-*β*-D-glucopyranoside (**F-21**), naringenin-7-*O*-[*β*-D-glucopyranosyl(1 → 3)-*α*-L-rhamnopyranosyl(1 → 2)-*O*-*β*-D-glucopyranoside (**F-22**), naringenin-7-*O*-*β*-D-xylopyranosyl-(1 → 6)-*β*-D-glucopyranoside (**F-23**) [20], naringoside (**F-24**) [33], naringenin (**F-25**) [5], (+)-4′-methylcatechin-7-*O*-*β*-D-glucopyranoside (**F-26**) [20], taxifolin (**F-27**) [38], cardamonin (**F-28**) [39], 4′,4′″,5,5″,7,7″-hexahydroxy-8,8″-biflavanonol (**F-29**) [39,40], 5,6,7,8,3′,4′-hexamethoxyflavone (**F-30**), 3,5,6,7,8,3′,4′-hexamethoxyflavone (**F-31**) [38], epicatechin-(5,6-bc)-4*β*-(*p*-hydroxyphenyl)-dihydro-2(3*H*)-pyranone (**F-32**), phloretin and (**F-33**) [41] are presented in Figure 5.

### 2.3. Polysaccharides

Natural carbohydrates called polysaccharides are present in many plants and can be used as a criterion to assess the quality of herbs that contain them [47,48]. Specifically, polysaccharides from *C. oleifera* fruits are important components found in the cake meal and the fruit shells.

These days, the extraction, separation, and purification of *C. oleifera* polysaccharides are the primary areas of chemical research. As studies into the fundamental structure of these polysaccharides have progressed, some insight into their glycosyl composition has emerged. The main monosaccharides that have been identified are rhamnose, glucose, glucuronic acid, galacturonic acid, arabinose, xylose, mannose, galactose, and fucose. The typical relative molecular mass of the polysaccharides ranges from 10^3^ to 10^7^ Da [49,50]. The chemical structural characteristics of those that have been found thus far are shown in Table 3.

### 2.4. Lignans

Fewer lignan compounds were isolated from the fruits of *C. oleifera*. Four lignans—(-)-pinoresinol-4-*O*-*β*-D-glucopyranoside, methylpinoresinol, (-)-pinoresinol, and (-)-pinoresinol diglucoside—were isolated from *C. oleifera* seed cake by Zhu et al. [44]. Cheng [39] isolated some lignans from the shells of *C. oleifera* fruits, including (+)-epipinoresinol, (1*S*,2*R*,5*S*,6*R*)-2-(4-hydroxyphenyl)-6-(3-methoxy-4-hydroxyphenyl)-3,7-dioxabicyclo [0,3] octane, syringaresinol, salicifoliol, (-)-secoisolariciresinol, arctigenin, (+)-isolariciresinol, pinnatifidanin B VI, pinnatifidanin B V, dihydrodehydrodiconiferl alcohol. (-)-isolariciresinol-9′-methyl succinate and (-)-isolariciresinol-9-methyl succinate, two new aryltetralin-type lignans were isolated from *C. oleifera* fruit husk [59]. (7*S*,8*S*)-3-methoxy-3′,7-epoxy-8,4′-oxyneoligna-4,9,9′-triol, massonianoside E, isolariciresinol-9-*O*-xyloside, nudiposide, 5′-methoxy-isolarchitin-9′-*O*-*β*-D-xyloside, aviculin, davidioside A, (7*R*,8*R*)-4-*O*-(glycer-2-yl)-7,9,9′-trihydroxy-3,3′-dimethoxy-8-*O*-4′-neolignan from the fruit hull of *C. oleifera* [48]. Sesamin and 2,5-bis-benzo[1,3]dioxol-5-yl-tetrahydro-furo [3,4-d][1,3]-dioxine were isolated from the methanol extract of tea seed oil [60]. 

### 2.5. Phytosterols

Phytosterols are naturally found in various parts of plants, including roots, stems, leaves, fruits, and seeds, and can be classified into three major categories: 4-methylsterols, 4,4′-dimethylsterols, and sterols without methyl substitutions. *C. oleifera* fruit contains geranyl linalool, ergosterol, *β*-amyrin, lanosterol [61], 3*α*-spinasterol, ergosta-4,6,8(14),22-tetraen-3-one [37], *β*-sitosterol, stigmast-7-en-3-ol [62]. 

### 2.6. Others

Fatty acids, phenylpropyl compounds, anthraquinones, volatile oils, alkaloids, and other bioactive substances have also been isolated from *C. oleifera* fruits. The main fatty acid constituents in its seeds are stearic acid, palmitic acid, oleic acid, and linoleic acid. Other components include palmitic acid, arachidic acid, α-linolenic acid, and others. Unsaturated fatty acids can make up more than 90% of the total. Oleic acid (C18:1), which ranged from 70.21% to 85.23%, palmitic acid (C16:0), which ranged from 6.93% to 13.89%, and linoleic acid (C18:2), which ranged from 5.02% to 14.26%, were the most prevalent fatty acids (FA) [63]. The fruit shells of *C. oleifera* were used to separate quinone components, including emodin, 6-ethyl-5-hydroxy-2, 7-dimethoxy-1, 4-naphthoquinone, and ω-hydroxyemodin [38]. Additionally, phenolic substances such as gallotannin, ellagitannin, and catechin were identified using HPLC–ESI–MS technology; oxalic acid, citric acid, acetic acid, malic acid, and succinic acid were found to be the major organic acids present [45].

## 3. Pharmacological Activities

Modern pharmacological studies have demonstrated that the extract of *C. oleifera* fruit has multiple pharmacological effects, including anti-tumor, anti-allergy, hypoglycemic, lipid-lowering, anti-inflammatory, antioxidant, and protective effects on the gastric mucosa.

### 3.1. Anti-Tumor Activity

A tumor is a mass formed by abnormal proliferation of cells within the body. This abnormal hyperplasia may be benign or malignant cancer. Cancer is recognized as the second leading cause of death globally, and the incidence of cancer continues to rise rapidly, imposing an escalating burden on global public health. Although chemotherapy remains the most commonly employed treatment for cancer, its efficacy is often constrained by the development of drug resistance and significant adverse effects [64]. Numerous natural compounds have been identified to influence cancer cell apoptosis and exhibit anticancer properties through multi-target synergistic mechanisms.

**Figure 6:**
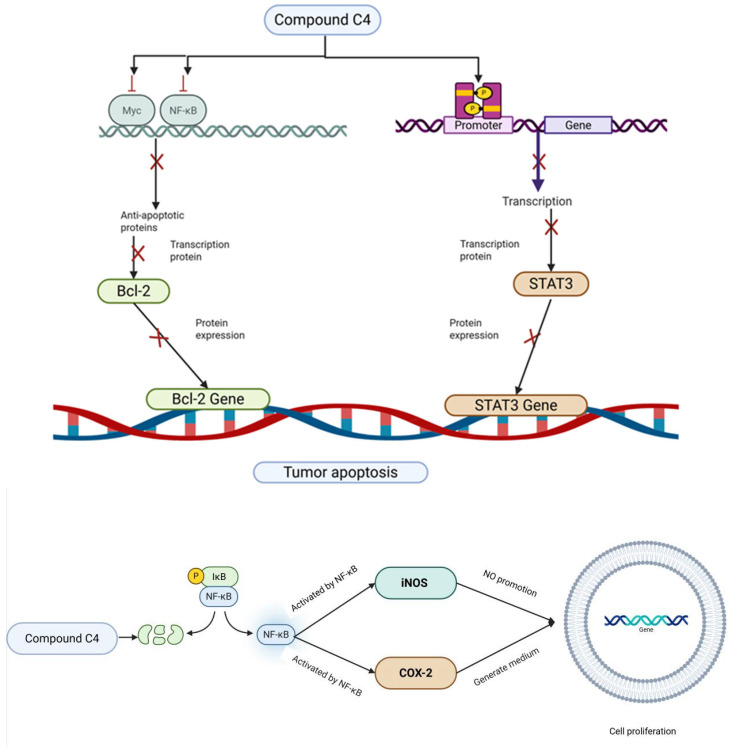
The mechanisms of action of compound C4 against cancer.

Previous studies have shown *C. oleifera* saponins exhibit significant anti-tumor effects both in vivo and in vitro, exerting direct cytotoxic actions and inhibiting the proliferation of tumor cells (Table 4). The cytotoxicity of oleiferasaponin B_1_ and B_2_ was evaluated in four human carcinoma cell lines: A549, SK-OV-3, SK-MEL-2, and HCT15. Both of them exhibited significant cytotoxic activity with IC_50_ values of 18.5 μM, 11.3 μM, 13.9 μM, and 1.6 μM; and IC_50_ values of 8.4 μM, 6.3 μM, 9.2 μM, and 0.8 μM [24]. Oleiferasaponin C_1_, C_2_, and camelliasaponin B_1_ showed significant cytotoxic activities against BEL-7402, BGC-823, MCF-7, HL-60, and KB, five human tumor cell lines [25]. Oleiferasaponin C_4_~C_6_ from the seeds of *C. oleifera* can inhibit proliferation through inducing cell-cycle arrest and apoptosis in human cancer cell lines (BEL-7402, BGC-823, MCF-7, HL-60, and KB) in vitro [26,27]. Oleiferasaponins D_1_ and D_2_ exhibited cytotoxic activity against five human cancer cell lines (HCT-116, HepG2, BGC-823, NCI-H1650, and A2780), with IC_50_ values ranging from 3.31 to 10.23 μM. Oleiferasaponins D_3_~D_5_ showed moderate cytotoxic activities toward the tested cell lines [28]. C4 exhibits the strongest anticancer activity among the five cell lines, as evidenced by its ED_50_ value, which varies from 1.5 to 11.3 μM against Huh-7, HepG2, HeLa, A549, and SGC7901. This value is comparable to that of cisplatinum (CDDP) in these cell lines. Compound C4 may increase cell proliferation via the NF-B/iNOS/COX-2 pathway and induce apoptosis via the Bcl-2/Caspase-3 and JAK2/STAT3 pathways [31] (Figure 6). Bioactivity evaluations demonstrated that oleiferasaponins G_6_~G_9_, as well as the total saponin, exhibited significant proliferative inhibitory activity against HCT-116, HL-60, and HepG2 cell lines [32].

Total saponins of *C. oleifera* (TSSC) have an inhibitory effect on the growth of solid tumors of H22 liver cancer in mice. TSSC can promote the apoptosis of H22 by up-regulating the expression of Bax protein and down-regulating the expression of Bcl-2 protein simultaneously [65]. In a recent study, it was demonstrated that the ethanol-soluble acidic components of *C. oleifera* cake exhibited anti-tumor activities both in vitro and in vivo through regulated cell death, which involved mechanisms of apoptosis, pyroptosis, and ferroptosis. Moreover, the combination of ESAC and PD-1 inhibitors showed greater efficacy than either treatment alone, promoting pyroptosis through the activation of the NLRP3/Caspase-1/GSDMD signaling pathway [66].

With an IC_50_ value of 5.826 μg/mL, the crude polysaccharides had substantial anti-proliferative activity against HepG2 cells [67]. On normal IAR20 cells, however, the inhibitory rate was 0.61%, suggesting low toxicity. The anticancer potential is further supported by in vivo research; in BALB/C mice, SCP1 demonstrated an 85.6% inhibition rate against Sarcoma 180 solid tumors at a dose of 40 mg/kg [68].

### 3.2. Antioxidant Activity

An imbalance between the body’s oxidation and antioxidation processes is known as oxidative stress, and it is thought to be a major contributing cause to aging and disease. It can cause neutrophils to infiltrate and induce inflammation, increase protease secretion, and produce a large number of oxidation intermediate products, which can lead to an excess of reactive oxygen species and other substances, and harm cells and tissues [69,70]. Polyphenolic chemicals and unsaturated fatty acids are abundant in *C. oleifera oil*. Tea polyphenols and oleic acid are strong natural antioxidants that have been shown to be highly effective in scavenging free radicals in the body [41,71].

Studies have shown that polyphenols extracted from seed cake can alleviate H_2_O_2_-induced oxidative stress in cells by modulating SOD activity, reducing the levels of MDA and ROS, suppressing apoptosis, and inhibiting the activation of the NF-κB signaling pathway [72]. 

Out of the five solvent extracts, the methanol extract of tea seed oil exhibited the highest yield and the strongest antioxidant activity based on DPPH scavenging activity and Trolox equivalent antioxidant capacity [60]. ABTS, DPPH, and OH radicals were all effectively scavenged by the polysaccharide derived from *C. oleifera* seed cakes, with IC_50_ values of 2.94, 2.24, and 5.09 mg/mL, respectively [73]. Because of its greater ability to donate hydrogen, COP-E showed the most positive antioxidant impact among the studied polysaccharides COP-H, COP-U, COP-E, and COP-A [52]. SCP-a demonstrated more ABTS scavenging activity than SCP-b and SCP-c at the same dose. Its compact, dense, and helical surface shape, along with the higher galactose content in its monosaccharide composition, may be responsible for this increased activity. In contrast, SCP-c demonstrated superior metal chelating ability, which could be attributed to its relatively higher uronic acid and sulfate content [56]. 

*Bacillus subtilis* was utilized for the submerged fermentation of camellia seed cake. The antioxidant capacity of the resulting product (CSCH) was evaluated using DPPH, ABTS, and hydroxyl radical scavenging assays. The results indicated that CSCH has the potential to be developed as a novel, fully natural antioxidant and anti-tyrosinase agent [74].

### 3.3. Hypolipidemic Activity

Elevated blood lipid levels are a defining feature of hyperlipidemia, a condition that significantly increases the risk of a number of illnesses, most notably heart disease and stroke. Promising substitutes for traditional lipid-lowering drugs, which have a long list of side effects, are phytochemical substances [75]. 

On HepG2 cell lines, oleiferasaponin A_2_ demonstrated anti-hyperlipidemic activity by dramatically increasing the expression of ACOX-1, CPT-1, and ACOX-1 protein, while significantly downregulating the expression of SREBP-1c, FAS, and FAS protein, which inhibited fatty acid synthesis [23]. For eight weeks, the rats were given a basal diet, a high-fat diet, and a high-fat diet combined with a hot water extract of tea seed cake (CSE). The findings demonstrated that rats administered CSE had reduced levels of circulating leptin, lower levels of malondialdehyde and hydroxyproline in the liver, and decreased levels of epididymal and retroperitoneal fat compared to the high-fat diet group [76]. 

A high-fat diet, a high-fat diet with atorvastatin, a high-fat diet supplemented with *C. oleifera* polyphenols (2.5, 7.5, and 15 mL/kg), or a basal diet were given to the rats. The findings showed that the high-fat diet combined with polyphenol or atorvastatin treatment decreased body weight and the liver-to-body weight ratio. The activities of alanine aminotransferase and aspartate aminotransferase were also reduced, as were the levels of total cholesterol, triglycerides, and low-density lipoprotein cholesterol. High-density lipoprotein cholesterol, on the other hand, was elevated. Genes linked to hepatic lipid metabolism, such as ACAT1, DGAT2, FAS, and SREBP, showed markedly decreased relative expression levels [77].

Camellia oil may elevate serum oleic acid levels and reduce body weight and BMI in individuals with hypertriglyceridemia who maintain stable dietary intake and physical activity [78]. Consuming camellia oil has been demonstrated to influence inflammatory indicators and oxidative stress in women with hypercholesterolemia. A diet high in tea oil has been shown to lower blood levels of malondialdehyde. Low-density lipoprotein cholesterol (LDL-C), malondialdehyde (MDA), and C-reactive protein (CRP) are examples of inflammatory indicators that can lower the risk of cardiovascular diseases [79]. 

### 3.4. Hypoglycemic Activity

Hyperglycemia, hyperlipidemia, and hepatic steatosis are among the signs of type 2 diabetic mellitus (T2DM), a metabolic disorder. Statistics show that 463 million people worldwide have diabetes, with type 2 diabetes accounting for the great majority of cases. Some complications are brought on by this illness, which not only affects blood sugar levels but also poses a major threat to life [80].

Oleiferasaponin A_1_, which may have hypoglycemic properties, protected pancreatic cell lines against the harm that comes from too much glucose. Oleiferasaponin A_1_ prevented the damage caused by excessive glucose and increased insulin expression in RIN-m5f cells [21]. It was discovered that the polysaccharide CCP raises the relative glucose consumption rate in HepG2 cells in a dose-dependent manner within the range of 0.125–0.500 mg/mL [50]. The fruit hull also contained substantial amounts of two polysaccharides, CFPB and CFPA-3, which were isolated and showed dose-dependent inhibition of α-glucosidase activity with IC50 values of 11.80 and 10.95 μg/mL, respectively [81].

In streptozotocin-induced diabetic mice, both crude and purified polysaccharides at a dose of 200 mg/kg/d significantly alleviated various symptoms, reduced the levels of MDA, and enhanced the activities of glutathione peroxidase (GSH-Px), catalase (CAT), and superoxide dismutase (SOD) [53]. Further animal experiments indicated that administration of polysaccharides SCP-1 and SCP-2 at a dose of 400 mg/(kg·d) decreased plasma glucose levels in streptozotocin-induced diabetic mice by 46.83% and 33.00%, respectively. These polysaccharides also elevated the activities of GSH-Px, CAT, and SOD levels, while reducing MDA content in hyperglycemic mice [55].

### 3.5. Anti-Inflammatory Activity

Excessive free radicals, which are created when the body’s metabolism and biochemical reactions are out of balance, can harm biological macromolecules like DNA, proteins, and mitochondria as well as cell structures. This can result in a number of diseases, including cardiovascular and inflammatory conditions. Thus, controlling genes to limit the overproduction of free radicals is crucial for apoptosis and cell division as well as preserving human health [82].

According to pharmacological research, phenolic substances, certain triterpenoids, and *C. oleifera* seed extract (camellia oil) all have strong anti-inflammatory properties [83]. Significant analgesic and anti-inflammatory properties are exhibited by the diflavonoids that were extracted from the shells of *C. oleifera* fruits. The findings of the mouse writhing experiments with hot plates and acetic acid suggested that this diflavonone compound could significantly lower serum MDA levels, boost SOD and GSH-Px activities, and dose-dependently reduce rat foot swelling caused by carrageenan and mouse ear inflammation caused by croton oil [41]. The ethanol extract of *C. oleifera* seeds effectively inhibits Drosophila enteritis. The chemicals with comparatively high antienteritis activity were identified by ultra-high performance liquid chromatography-tandem mass spectrometry as bruceine B, miltirone, 8-geranyloxypsoralen, cedrelone, wighteone, kaempferitrin, and kaempferol-3-*O*-rutinoside [84].

The hydrolyzed sasanquasaponins extracted from the defatted seeds of *C. oleifera* exhibited significant anti-inflammatory and analgesic effects. These compounds effectively alleviated carrageenan-induced paw edema in rats and soybean oil-induced ear inflammation in mice. Furthermore, they were found to reduce serum MDA levels while simultaneously increasing the levels of SOD and GSH-Px. Additionally, these saponins demonstrated the ability to inhibit the expression of pro-inflammatory cytokines, including interleukin-1β (IL-1β), tumor necrosis factor-α (TNF-α), and prostaglandin E2 (PGE2) [85].

### 3.6. Neuroprotective Activity

A progressive, late-onset condition known as neurodegeneration is typified by loss or degeneration of neurons as well as cognitive and motor deficits. Its fundamental mechanics are yet not fully understood. Oxidative stress has been the subject of numerous investigations, as it plays a part in the series of events that culminate in neurodegenerative pathology [86].

Camellia oil contains unsaturated fatty acids, squalene, polyphenols, and other bioactive compounds. Its unsaturated fatty acids, such as oleic acid, can reduce the production of Aβ in the brain tissue of Alzheimer’s disease (AD) mice and inhibit the deposition of amyloid plaques caused by abnormal accumulation of Aβ [87]. It has been demonstrated that camellia oil regulates the metabolism of aspartic acid, increases the level of N-acetyl-L-aspartic acid in mice, enhances the brain’s energy supply, and alleviates the symptoms of AD [88]. A previous report demonstrated that camellia oil may reverse AD-related brain pathology by alleviating memory impairments, enhancing learning ability, increasing antioxidant activity, modulating the expression of immune-related cytokines, promoting autophagy, and improving the composition of gut microbiota in rats treated with aluminum chloride. These findings suggest that camellia oil may mitigate the pathophysiological progression of AD through mechanisms involving the microbiome-gut–brain axis [89]. Camellia oil may ameliorate Aβ_25–35_-induced memory impairment in mice by modulating immune cell activity and neuroinflammation through the PPARs signaling pathway, which in turn influences gut microbiota composition and lipid metabolism [90].

The sapogenin from sasanqua saponin hydrolysis and its amination derivative increase dopamine levels in the substantia nigra and striatum, increase the number of tyrosine hydroxylase-positive cells, reduce neuroinflammation and behavioral deficits, and protect against Parkinson’s disease in MPTP-treated mice. These compounds appear to shield dopaminergic neurons via anti-neuroinflammatory effects and dopamine receptor activation, with aminated derivatives showing greater efficacy [29]. Treatment with oleiferasaponin A_1_ at concentrations of 5, 25, and 125 μM significantly increased cell viability, indicating its cytoprotective effect against H_2_O_2_-induced damage [22].

While increasing superoxide dismutase activity, tyrosine hydroxylase expression, and dopamine and acetylcholine levels in a dose-dependent way, iron-sapogenin nanoparticles can ameliorate behavioral problems and lower malondialdehyde levels in the mouse brain. Iron-sapogenin nanoparticles have much better therapeutic benefits than sapogenin by itself [91]. Through the coordination of zinc with sapogenin, the nanoparticle promotes electron transport among atoms, increasing DPPH radical scavenging activity. Zinc-sapogenin administered intraperitoneally has been shown to improve antioxidant capacity, raise dopamine and acetylcholine levels in the brain, and reduce behavioral abnormalities and neuronal damage in mice induced by rotenone neurotoxicity [92].

### 3.7. Antimicrobial Activity

The health of people, animals, and the environment is seriously threatened by the global spread of antimicrobial resistance (AMR). Antimicrobial resistance has been accelerated by the widespread usage and incorrect application of antimicrobial drugs, despite the fact that they have revolutionized modern medicine [93]. As a result, natural antibacterial agents derived from plants have drawn more interest as possible substitutes.

Camelliasides A and B, as well as a saponin mixture containing camelliasaponin B1, demonstrate the ability to reduce *Rhizoctonia solani* Kühn AG-4 infection in cabbage seedlings and inhibit the growth of the pathogen on potato dextrose agar plates [21]. Two compounds from *C. oleifera* fruit hull, epicatechin-(5,6-bc)-4*β*-(p-hydroxyphenyl)-dihydro-2(3H)-pyranone and 2-*O*-(3,4-dihydroxybenzoyl)-2,4,6-trihydroxyphenylmthylacetate, show good anti-respiratory syncytial virus activity. The IC_50_ values are 9.67 ± 0.68 and 21.53 ± 2.54, respectively [41].

From camellia oil, Akihisa et al. extracted seven triterpenoids (1–7) and assessed their capacity to prevent Raji cells from producing Epstein–Barr virus early antigens (EBV-EA) in response to stimulation. With IC_50_ values ranging from 277 to 420 mol TPA/32 pmol TPA, compounds 5–7 showed notable inhibitory efficacy against EBV-EA induction [94]. The minimum inhibitory concentration (MIC) values of tea oil extracted using organic solvents against *Escherichia coli*, *Staphylococcus aureus*, and yeast were all 100 mg/mL, with inhibitory effects increasing as the concentration increased. For pressed tea oil, the MIC value against *E. coli* was 100 mg/mL, while those against *S. aureus* and yeast were 200 mg/mL. Inhibition by pressed tea oil also exhibited a concentration-dependent effect. In contrast, no antimicrobial activity was detected against *Penicillium*, green mold, or *Bacillus subtilis* [95]. It has been reported that four samples of *C. oleifera* seed oil at different refining stages, as well as their methanol extracts, have certain inhibitory effects on *E. coli*, *S. aureus*, *Pseudomonas aeruginosa*, and *Candida albicans*. The primary antibacterial active constituents are speculated to include catechin derivatives, chlorogenic acid, puerarin, carotene, α-tocopherol, and 3-*p*-coumaryl quinic acid [96].

### 3.8. Other Activity

The components of *C. oleifera* fruit also have the functions of regulating intestinal flora, protecting gastric mucosa, immunomodulation, and so on. 

Jin et al. [57] obtained two polysaccharides (CCPA and CCPB) from tea seed cakes. Animal experiments demonstrated that these polysaccharides could modulate gut microbiota composition and enhance microbial diversity.

The phagocytic activity of primary peritoneal macrophages and RAW 264.7 macrophages was markedly increased by camellia oil. It also decreased the generation of nitric oxide (NO) in BALB/c mice and RAW 264.7 cells. Additionally, camellia oil significantly increased the production of IL-10 by primary splenocytes. 

In addition to increasing the mRNA expression of heme oxygenase-1 (HO-1), GSH-Px, and SOD, treating Int-407 cells with 50–75 μg/mL of camellia oil also increased the secretion of vascular endothelial growth factor (VEGF) and PGE2, strengthening the mucosal defense against gastrointestinal oxidative injury. Additionally, pretreatment with camellia oil (2 mL/kg/d) before ketoprofen administration (50 mg/kg/d) in Sprague Dawley rats inhibited the expression of the cyclooxygenase-2 (COX-2) protein, decreased the production of NO and IL-6, restored compromised antioxidant systems, and lessened oxidative damage in the gastrointestinal mucosa [97].

Camellia oil significantly enhanced phagocytic activity in both RAW 264.7 macrophages and primary peritoneal macrophages. Additionally, it reduced NO production in RAW 264.7 cells and BALB/c mice. Moreover, camellia oil markedly stimulated IL-10 production in primary splenocytes. These findings suggest that camellia oil, which is rich in oleic acid, can potently promote the CD19+-mediated humoral immune response [98].

## 4. Conclusions

**Figure 7:**
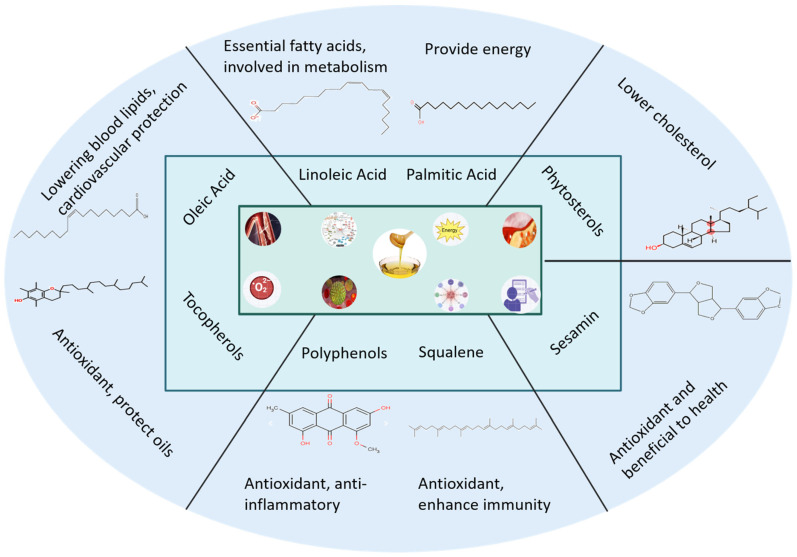
The main components and their pharmacological activities of camellia oil.

*C*. *oleifera*, a Chinese endemic woody oil crop cultivated for over 2000 years, yields seed oil rich in unsaturated fatty acids. As a traditional medicinal plant, it demonstrates anti-inflammatory, antioxidant, and lipid-regulating properties, showing promise for pharmaceutical and functional product development. China currently has a cultivation area of approximately 30,000 m^2^ for *C. oleifera*, with an annual production of one million tons of Camellia oleifera seeds and 270,000 tons of oil. As a pure, natural, woody, edible, and healthy vegetable oil (Figure 7), tea oil is promoted by the Chinese government and is the first plant-based edible oil recommended by the Food and Agriculture Organization of the United Nations.

The utilization of seed oil is currently the main focus of research and development activities on this plant, although the utilization of by-products like fruit shells and cake meal is still in its infancy. There is a serious risk of environmental contamination because these processing leftovers are often discarded as waste after manufacturing. To solve this problem, the primary phytochemical components isolated from *C. oleifera* fruits and their pharmacological effects were covered in this review. In terms of the number of isolated compounds, triterpene saponins are the most numerous, followed by flavonoids and polysaccharides. In the meantime, *C. oleifera*’s constituents have outstanding pharmacological properties and can serve as lead compounds for the creation of novel medications. The primary focus of current research on the therapeutic regulation of these substances is their pharmacological effects and underlying mechanisms. Future studies should prioritize clinical application research to bridge the gap between preclinical findings and therapeutic practice. In addition, some evidence suggests that *C. oleifera*’s bioactive constituents may modulate gut microbiota composition. As compound efficacy is intrinsically linked to bioavailability and host-microenvironment interactions, systematic investigations into their microbiota-modulating effects are warranted. 

In conclusion, as mentioned above, it offers valuable insights for further research on the health benefits of this plant and suggests new potential approaches for the large-scale utilization of *C. oleifera* in disease prevention and treatment.

## Figures and Tables

**Table 1:** Triterpenoids and triterpenoid saponins from *C. oleifera* fruit.

No.	Compound Name	Chemical Formula	R_1_	R_2_	R_3_	R_4_	R_5_	R_6_
**1** [20]	21-*O*-angeloyl-28-*O*-acetyltheasapogenol E 3-*O*-[*β*-D-galactopyranosyl(1 → 2)] [*β*-D-xylopyranosyl (1 → 2)-*α*-L-arabinopyranosyl (1 → 3)]-methyl *β*-D-glucopyranosiduronate	C_60_H_92_O_27_	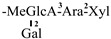	CHO	OH	OAc	OH	OAng
**2** [6]	camelliasaponin B_1_	C_58_H_89_O_26_	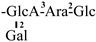	CHO	OH	OH	OH	OAng
**3** [21]	camelliasaponin B_2_	C_58_H_90_O_26_	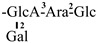	CHO	OH	OH	OH	OTig
**4** [22]	oleiferasaponin A_1_	C_59_H_92_O_26_	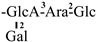	CHO	OH	OH	OHex	H
**5** [23]	oleiferasaponin A_2_	C_63_H_99_O_28_	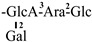	CHO	OH	OH	OIso	OAng
**6** [21]	oleiferasaponin A_3_	C_67_H_96_O_28_	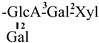	CHO	OH	OH	OCin	OAng
**7** [24]	oleiferasaponin B_1_	C_58_H_90_O_26_	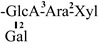	CHO	OH	OH	OTig	CH_2_OH
**8** [24]	oleiferasaponin B_2_	C_61_H_90_O_24_	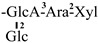	CHO	OH	OH	OCin	H
**9** [25]	oleiferasaponin C_1_	C_59_H_91_O_26_	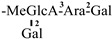	CHO	OH	H	OAng	H
**10** [25]	oleiferasaponin C_2_	C_60_H_95_O_26_	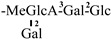	CH_3_	OH	H	OAng	H
**11** [25]	oleiferasaponin C_3_	C_63_H_91_O_26_	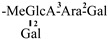	CHO	OH	OCin	OH	H
**12** [26]	oleiferasaponin C_4_	C_60_H_94_O_27_	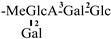	CHO	OH	OH	OAng	H
**13** [26]	oleiferasaponin C_5_	C_54_H_84_O_22_	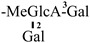	CHO	OH	OH	OAng	H
**14** [27]	oleiferasaponin C_6_	C_65_H_94_O_28_	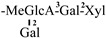	CHO	OH	OH	OCin	OAc
**15** [28]	oleiferasaponin D_1_	C_58_H_91_O_25_	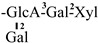	CH_3_	OH	OH	OAng	H
**16** [28]	oleiferasaponin D_2_	C_58_H_89_O_26_	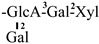	CHO	OH	OH	OAng	H
**17** [28]	oleiferasaponin D_3_	C_58_H_89_O_26_	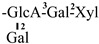	CHO	OH	OH	OTig	H
**18** [28]	oleiferasaponin D_4_	C_58_H_91_O_26_	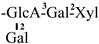	CHO	OH	OH	OMB	H
**19** [28]	oleiferasaponin D_5_	C_58_H_91_O_27_	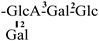	CHO	OH	OH	OTig	H
**20** [29]	sasanqua saponin	C_30_H_48_O_5_	H	CHO	OH	OH	OH	H
**21** [30]	gordonoside R	C_62_H_96_O_26_	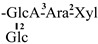	CH_3_	OH	OH	OAng	OAng
**22** [30]	gordonoside Q	C_62_H_96_O_26_	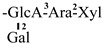	CH_3_	OH	OH	OAng	OAng
**23** [31]	C1	C_35_H_54_O_6_	H	CHO	OTig	OH	OH	H
**24** [31]	C2 (camelliagenin A 16-tiglate)	C_35_H_56_O_5_	H	CH_3_	OTig	OH	OH	H
**25** [31]	C3 (camelliagenin A 22-tiglate)	C_35_H_56_O_5_	H	CH_3_	OH	OH	OTig	H
**26** [31]	C4	C_47_H_70_O_14_	MeGlcA	CH_3_	OH	OH	OTig	OTig
**27** [32]	oleiferasaponin G_1_	C_58_H_90_O_26_	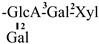	CHO	OTig	OH	OH	H
**28** [32]	oleiferasaponin G_2_	C_59_H_92_O_27_	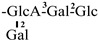	CHO	OTig	OH	OH	H
**29** [32]	oleiferasaponin G_3_	C_59_H_92_O_27_	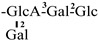	CHO	OAng	OH	OH	H
**30** [32]	oleiferasaponin G_4_	C_59_H_92_O_27_	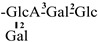	CHO	OMB	OH	OH	H
**31** [32]	oleiferasaponin G_5_	C_58_H_90_O_26_	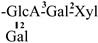	CHO	OAng	OH	OH	H
**32** [32]	oleiferasaponin G_6_	C_60_H_92_O_28_	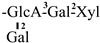	CHO	OH	OH	OAng	OAc
**33** [32]	oleiferasaponin G_7_	C_59_H_92_O_27_	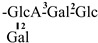	CHO	OH	OH	OTig	H

Note: “→” The arrows denote glycosidic linkages between monosaccharide residues.

**Table 2:** Flavonoids and their glycosides from *C. oleifera* fruit.

No.	Compound Name	Chemical Formula	R_1_	R_2_	R_3_	R_4_
**F-1** [35]	kaempferol	C_15_H_10_O_6_	H	H	H	H
**F-2** [36]	quercetin	C_15_H_10_O_7_	H	H	OH	H
**F-3** [36]	rutin	C_27_H_30_O_16_	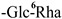	H	OH	H
**F-4** [36]	kaempferol-3-*O*-*β*-D-glucopyranosyl-(1 → 2)-*α*-L-arabinopyranoside	C_26_H_30_O_16_	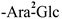	H	H	H
**F-5** [35]	kaempferol-3-*O*-*α*-L-rhamnopyranosyl-(1 → 6)-*β*-D-glucopyranoside	C_27_H_30_O_15_	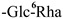	H	H	H
**F-6** [35]	kaempferol-3-*O*-[2-*O*-*α*-L-rhamnopyranosyl-6-*O*-*β*-D-glucopyranosyl]-*β*-D-glucopyranoside	C_33_H_40_O_20_	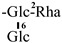	H	H	H
**F-7** [35]	kaempferol-3-*O*-[2-*O*-*β*-D-xylopyranosyl-6-*O*-*α*-L-rhamnopyranosyl]-*β*-D-glucopyranoside	C_32_H_38_O_19_	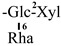	H	H	H
**F-8** [35]	kaempferol-3-*O*-[2-*O*-*α*-L-rhamnopyranosyl-6-*O*-*β*-D-glucopyranosyl]-*β*-D-glucopyranoside	C_32_H_38_O_19_	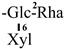	H	H	H
**F-9** [36]	kaempferol-3-*O*-[2-*O*-*β*-D-galacotopyranosyl-6-*O*-*α*-L-rhamnopyranosyl]-*β*-D-glucopyranoside	C_33_H_40_O_20_	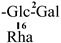	H	H	H
**F-10** [42]	kaempferol-3-*O*-[2-*O*-*β*-D-glucopyranosyl-6-*O*-*α*-L-rhamnopyranosyl]-*β*-D-glucopyranoside	C_33_H_40_O_20_	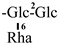	H	H	H
**F-11** [20,43]	kaempferol 3,7-*O*-bis-*α*-L-rhamnopyranoside (kaempferitrin)	C_27_H_30_O_14_	Rha	Rha	H	H
**F-12** [43]	kaempferol-3-*O*-*β*-D-glucopyranoside	C_21_H_20_O_11_	Glc	H	H	H
**F-13** [43]	isorhamnetin	C_16_H_12_O_7_	H	H	H	OCH_3_
**F-14** [44]	quercetin-3-*O*-*β*-D-glucopyranoside	C_21_H_20_O_12_	Glc	H	OH	H
**F-15** [44]	quercetin-3-*O*-*β*-D-glucuronide	C_21_H_18_O_13_	GlcA	H	OH	H
**F-16** [44]	kaempferol-3-*O*-*β*-D-glucuronide	C_21_H_18_O_12_	GlcA	H	H	H
**F-17** [45]	kaempferol 3-*O*-[*α*-L-rhamnopyranosyl-(1 → 6)-*β*-D-glucopyranosyl]-7-*O*-*β*-D-glucopyranoside	C_33_H_40_O_20_	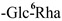	Glc	H	H
**F-18** [45]	kaempferol 3-*O*-[*β*-D-glucopyranosyl-(1 → 4)-*α*-L-rhamnopyranosyl]-7-*O*-*α*-L-rhamnopyranoside	C_33_H_40_O_19_	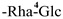	Rha	H	H
**F-19** [46]	kaempferol-3-*O*-[*β*-D-glucopyranosyl-(1 → 3)-*α*-L-rhamnopyranosyl-(1 → 6)-*O*-*β*-D-galactopyranoside	C_33_H_40_O_20_	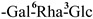	H	H	H

Note: “→” The arrows denote glycosidic linkages between monosaccharide residues.

**Table 3:** Polysaccharides from *C. oleifera* fruit.

No.	Polysaccharides	The Types and Proportions of Monosaccharides	M.W. (Da)	Total Sugar Content
**1** [49]	CCP	Xyl:GlcA:Gal:Man =10.9:4.4:2.6:1.8	4736	–
**2** [51]	–	Glc:Rha:Man:GalA:Gal:Xyl:Ara = 1.0:0.40:0.28:0.09:0.17:0.10:0.07	–	81.20%
**3** [50]	CSP-1	Rha:Ara:Gal:Glc:Xyl:Man:GalA:GlcA = 0.04:0.16:0.10:0.34:0.02:0.02:0.27:0.02	8.90 × 10^4^, 13.51 × 10^4^	75.93%
**4** [50]	CSP-2	Rha:Ara:Gal:Glc:Xyl:Man:GalA:GlcA = 0.04:0.23:0.18:0.20:0.03:0.01:0.03:0.04	9.92 × 10^4^	69.22%
**5** [50]	CSP-3	Rha:Ara:Gal:Glc:Xyl:Man:GalA:GlcA = 0.03:0.12:0.07:0.20:0.01:0.01:0.02:0.01	1.83 × 10^5^	80.61%
**6** [50]	CSP-4	Rha:Ara:Gal:Glc:Xyl:Man:GalA:GlcA = 0.05:0.18:0.08:0.15:0.01:0.02:0.03:0.01	3.61 × 10^5^, 7.426 × 10^5^	75.93%
**7** [52]	COP-H	Glc:Gal:Man:Rha:Ara:Fuc = 46.55:18.89: 15.33:8.37:1.11:9.75	3.94 × 10^5^	–
**8** [52]	COP-U	Glc:Gal:Man:Rha:Ara:Fuc = 63.06:8.28:9.58:9.04:3.95:6.09	2.97 × 10^5^	–
**9** [52]	COP-E	Glc:Gal:Man:Rha:Ara:Fuc = 43.32:11.77: 14.45:5.97:5.11:19.37	4.62 × 10^5^	–
**10** [52]	COP-A	Glc:Gal:Man:Rha:Ara:Fuc = 38.31:14.41: 19.00:12.96:3.29:12.04	4.29 × 10^5^	–
**11** [53]	SCFP	Rha:Xyl:Glc = 11.19:17.06:5.81	2.0121 × 10^7^	–
**12** [54]	COP-1	Man:Rha:GalA:Glc:Gal:Xyl:Ara = 2.16:1.67:2.34:78.55:8.97:1.39:4.91	7.90 × 10^3^	–
**13** [54]	COP-2	Man:Rha:GalA:Glc:Gal:Xyl:Ara = 2.73:2.38:2.84:72.65:10.82:1.85:6.75	3.60 × 10^4^	–
**14** [54]	COP-3	Man:Rha:GlcA:GalA:Glc:Gal:Xyl:Ara = 2.23:2.45:5.79:5.04:60.03:11.98:1.67:10.81	8.30 × 10^4^	–
**15** [54]	COP-4	Man:Rha:GlcA:GalA:Glc:Gal:Xyl:Ara = 1.58:2.15:10.29:2.96:56.80:11.20:1.14:13.89	2.25 × 10^5^	–
**16** [55]	SCP-1	Man:Glc:Xyl = 1.77:0.93:1.00	7.16 × 10^6^	–
**17** [55]	SCP-2	Man:Rha:Glc:Xyl = 5.27:1.21:0.16:1.00	2.00 × 10^4^	–
**18** [56]	SCP-a	Man:Rha:Glc:Gal:Xyl = 1.00:16.82:19.80:103.46:41.96	8.26 × 10^4^	80.12%
**19** [56]	SCP-b	Man:Rha:Glc:Gal:Xyl:Ara = 9.08:1.00:1.00:1.35:0:1.51	2.05 × 10^7^	2.30%
**20** [56]	SCP-c	Man:Rha:Glc =35.74:1.00:5.26	1.44 × 10^7^	76.12%
**21** [57]	CCPA	GalA:Glc:Rha:Gal:Man:Ara = 16.27:16.89:16.02:17.32:14.28:19.22	7.02 × 10^4^	84.36%
**22** [57]	CCPB	GalA:Glc:Rha:Gal:Man:Ara = 6.02:18.88:14.38:20.49:17.79:22.44	2.46 × 10^6^	80.30%
**23** [58]	CFP-U	Man:Rha:GlcA:GalA:Glc:Gal:Xyl:Fuc = 0.05:0.14:005:1.00:0.05:0.30:0.35:0.41	9.16 × 10^4^	–

**Table 4:** Pharmacological activities of main saponin compounds from *C. oleifera* fruits.

No.	Chemical Constituents	Pharmacological Activities	Models	Mechanisms/Effects
**1** [24]	oleiferasaponin B_1_	anti-tumor activity	human tumor cell lines (A549, SK-OV-3, SK-MEL-2, and HCT15), in vitro	IC_50_ values of 18.5 μM (A549), 11.3 μM (SK-OV-3), 13.9 μM (SK-MEL-2), and 1.6 μM (HCT15)
**2** [24]	oleiferasaponin B_2_			IC_50_ values of 8.4 μM (A549), 6.3 μM (SK-OV-3), 9.2 μM (SK-MEL-2), and 0.8 μM (HCT15)
**3** [25]	oleiferasaponin C_1_	anti-tumor activity	human cancer cell lines (BEL-7402, BGC-823, MCF-7, HL-60, and KB), in vitro	IC_50_ values of 6.641 μM (BEL-7402), 12.766 μM (BGC-823), 5.567 μM (MCF-7), 7.930 μM (HL-60), 12.215 μM (KB)
**4** [25]	oleiferasaponin C_2_			IC_50_ values of 9.601 μM (BEL-7402), 13.558 μM (BGC-823), 11.223 μM (MCF-7), 4.590 μM (HL-60), 12.995 μM (KB)
**5** [25]	camelliasaponin B_1_			IC_50_ values of 17.649 μM (BEL-7402), 25.788 μM (BGC-823), 12.995 μM (MCF-7), 18.071 μM (HL-60), 16.544 μM (KB)
**6** [26]	oleiferasaponin C_4_	anti-tumor activity		IC_50_ values of 10.385 μM (BEL-7402), 11.242 μM (BGC-823), 15.094 μM (MCF-7), 6.489 μM (HL-60), 12.302 μM (KB)
**7** [26]	oleiferasaponin C_5_			IC_50_ values of 17.649 μM (BEL-7402), 25.788 μM (BGC-823), 12.995 μM (MCF-7), 18.071 μM (HL-60), 16.544 μM (KB)
**8** [27]	oleiferasaponin C_6_			IC_50_ values of 4.023 μM (BEL-7402), 6.001 μM (BGC-823), 9.016 μM (MCF-7), 1.876 μM (HL-60), 6.119 μM (KB)
**9** [28]	oleiferasaponins D_1_	anti-tumor activity	human tumor cell lines (HCT-116, HepG2, BGC-823, NCI-H1650, and A2780), in vitro	IC_50_ values of 8.33 μM (HCT-116), 6.11 μM (HepG2), 9.53 μM (BGC-823), 7.85 μM (NCI-H1650), 6.48 μM (A2780)
**10** [28]	oleiferasaponins D_2_			IC_50_ values of 10.23 μM (HCT-116), 8.52 μM (HepG2), 4.86 μM (BGC-823), 3.31 μM (NCI-H1650), 8.49 μM (A2780)
**11** [22]	oleiferasaponin A_1_	neuroprotective activity	PC12 cells injured by H_2_O_2_ at 5, 25, and 125 μM, in vitro	potentially prevent
[21]				
		hypolipidemic activity	RIN-m5f was injured by high glucose, in vitro	protecting pancreatic-cell lines from high-glucose damage, insulin levels of RIN-m5f cells ↑
**12** [23]	oleiferasaponin A_2_	hypolipidemic activity	on HepG2 cell lines, in vitro	SREBP-1c, FAS ↓, CPT-1, and ACOX-1 ↑
**13** [29]	Susanqua saponin	neuroprotective activity	injected with 20 mg/kg MPTP once every 2 h for three consecutive times, Kunming mice, in vivo	increase dopamine content in the striatum and tyrosine hydroxylase positive cells in the substantia nigra, and relieve inflammation and behavioral disorder
**14** [31]	C4	anti-tumor activity	human tumor cell lines (Huh-7, HepG2, Hela, A549, SGC7901, 239T, U251 and PAN02), in vivo	the ED_50_ values ranged from 1.5 to 11.3 μM p-mTOR, p-STAT3, and Bcl-2 ↓
			LPS-induced HepG2 cells, in vivo	β-catenin, Nrf2, HO-1 ↓ Caspase-3, TNF-α, NF-kβ, iNOS, and COX-2 ↑
**15** [32]	oleiferasaponin G_6_	anti-tumor activity	human tumor cell lines (HCT-116, HL-60, HepG2), in vitro	IC_50_ values of 1.200 μM (HCT-116), 3.515 μM (HL-60), and 2.518 μM (HepG2)
**16** [32]	oleiferasaponin G_7_			IC_50_ values of 1.208 μM (HCT-116), 2.938 μM (HL-60), and 2.711 μM (HepG2)
**17** [32]	oleiferasaponins D_2_			IC50 values of 1.264 μM (HCT-116), 2.775 μM (HL-60), and 1.204 μM (HepG2)
**18** [32]	oleiferasaponins D_3_			IC50 values of 1.181 μM (HCT-116), 5.596 μM (HL-60), and 2.704 μM (HepG2)

Note: The symbols ↓ and ↑ represent an increase and a decrease in the indicator value, respectively.

## Data Availability

Data are contained within the article.
